# Influence of Hydroxyapatite Coating for the Prevention of Bone Mineral Density Loss and Bone Metabolism after Total Hip Arthroplasty: Assessment Using ^18^F-Fluoride Positron Emission Tomography and Dual-Energy X-Ray Absorptiometry by Randomized Controlled Trial

**DOI:** 10.1155/2020/4154290

**Published:** 2020-02-22

**Authors:** Taro Tezuka, Naomi Kobayashi, Choe Hyonmin, Masatoshi Oba, Yushi Miyamae, Akira Morita, Yutaka Inaba

**Affiliations:** ^1^Department of Orthopedic Surgery, Yokohama City University, 3-9 Fukuura, Kanazawa-ku, Yokohama, Japan; ^2^Department of Orthopaedic Surgery, Yokohama City University Medical Center, 4-57 Urafunecho, Minami-ku, Yokohama, Japan

## Abstract

**Background:**

Hydroxyapatite- (HA-) coated implants tend to achieve good osteoinductivity and stable clinical results; however, the influence of the coating on the prevention of bone mineral density (BMD) loss around the implant is unclear. The purpose of this randomized controlled trial was to evaluate the effectiveness of HA-coated implants for preventing BMD loss and to determine the status of bone remodeling after total hip arthroplasty (THA), making comparisons with non-HA-coated implants.

**Methods:**

A total of 52 patients who underwent primary THA were randomly allocated to HA and non-HA groups. BMD was measured by dual-energy X-ray absorptiometry (DEXA) at 1 week postoperation to form a baseline measurement, and then 24 weeks and 48 weeks after surgery. The relative change in BMD was evaluated for regions of interest (ROIs) based on the Gruen zone classifications. ^18^F-fluoride positron emission tomography (PET) was performed at 24 weeks postsurgery, and the maximum standardized uptake values (SUV_max_) were evaluated in the proximal (HA-coated) and distal (non-HA-coated) areas in both groups.

**Results:**

There were significant differences in BMD loss in ROIs 3 and 6 (*p* = 0.03), while no significant difference was observed in ROI 7 at either 24 or 48 weeks postsurgery. There was no significant correlation between PET uptake and BMD (24 or 48 weeks) in either group.

**Conclusion:**

The influence of a HA coating in terms of BMD preservation is limited. No significant correlation was found between BMD and SUV_max_ measured by PET, either with or without the use of a HA coating.

## 1. Background

Total hip arthroplasty (THA) is recognized as achieving stable long-term clinical results and excellent patient satisfaction; as for tapered rectangular Zweymüller type, the long-term reported survival rates were 98% at 20 years by Kolb et al. or 95% at 18 years by Reigstad et al. regarded as almost satisfactory. However, despite the stable clinical results, several issues concerning radiographic assessment after THA remain unresolved [1]. One of the major issues on the femoral side is a decrease in bone mineral density (BMD) around the implant. Significant decreases in BMD in the proximal region of uncemented prosthetic femoral stems have been reported, especially in stems with metaphyseal fixation such as Zweymüller type [2–4]. While bisphosphonate has a significant effect in preventing this BMD loss after THA [5], there is controversy over the appropriateness of long-term bisphosphonate administration after THA [6]. Furthermore, less invasive surgery has been introduced and *a modified Zweymüller type* stem, SL-PLUS MIA (Smith and Nephew, Memphis, TN, USA), was developed for less invasive surgery with omission of the lateral wing. Using the SL-PLUS MIA stem also reduces the loss of bone tissue and soft tissue trauma; however, radiolucent lines (RLL) around the proximal femoral prosthesis have been reported [7, 8]. To prevent such RLL, the SL-PLUS MIA HA stem was introduced. Thus, the SL-PLUS MIA stems have a 0.05 mm hydroxyapatite (HA) layer coating on its proximal area. A histological evaluation of retrieved hydroxyapatite- (HA-) coated implants revealed that adding a HA coating to the implant surface had a positive effect on bone growth [9, 10]. Therefore, as this is still a subject of controversy, we asked if the use of a HA coating on stems is expected to have a measurable effect on the prevention of BMD loss around the prosthetic femoral stem the same as the osteointegration on the stem and the prevention of RLL. Previous studies focused mainly on radiographic evaluations [9]; however, positive evidence of the effectiveness of a HA coating for preventing BMD loss around implants is still limited [11, 12].

Recent developments in nuclear imaging technology for bone imaging have been remarkable, and ^18^F-fluoride positron emission tomography (PET) has emerged as a promising modality with which it is possible to evaluate osteoblastic activity [13]. Several previous studies utilized ^18^F-fluoride PET to evaluate the status of bone remodeling around femoral implants after THA [14–17]. Although the fundamental target of such PET imaging is not the evaluation of BMD, we hypothesized that there may be a correlation between PET uptake and BMD, and also that PET uptake may be influenced by the HA coating around an implant. Therefore, to study the relationship between PET uptake and BMD, we applied dual-energy X-ray absorptiometry (DEXA) to analyze BMD changes and ^18^F-fluoride PET to analyze the osteoblastic activity of periprosthetic tissue [18]. The primary goal of this study was to determine the effectiveness of HA coatings for preventing BMD loss, while a second outcome was the evaluation of the correlation between PET uptake and BMD.

## 2. Methods

**Figure 1:**
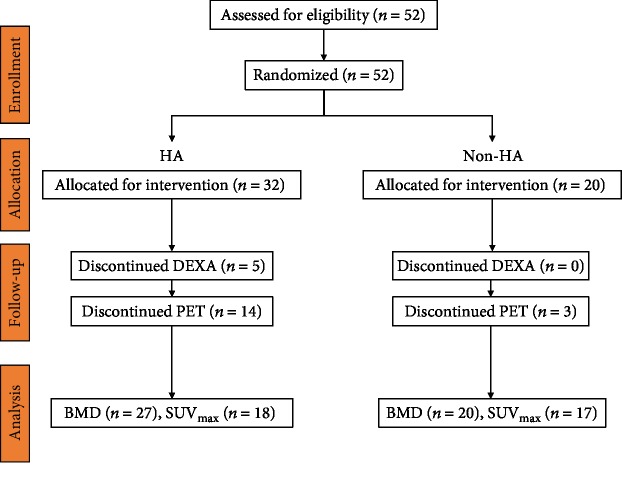
Flow chart of patient assignment. HA: hydroxyapatite.

This prospective randomized controlled trial was approved by the institutional review board of Yokohama City University (B181200022), and informed consent was obtained from each patient. The study took place at the Yokohama City University in Yokohama, Japan, from January 2006 to April 2007. A total of 52 subjects were randomly assigned to either a group implanted with SL-PLUS™ MIA stems with a HA coating or a group implanted with SL-PLUS MIA, non-HA-coated stems (Figure 1). All patients had no history of treatment with bisphosphonates or steroids which had influence on bone metabolism. Before starting to recruit, we did a power analysis with effect size of 0.4, alpha of 0.05, and 1-beta of 0.90; 52 should be appropriate to evaluate our primary endpoint, i.e., the BMD between 2 types of stems. For allocation of the participants, a computer-generated list of random numbers was used.  Table 1 shows the demographic data of each group. There were no statistically significant differences between the demographics of the HA and non-HA groups. After patient drop out due to failure to undergo PET or DEXA, BMD was evaluated in 47 subjects and PET in 35.

This study was registered into the university hospital medical information network (UMIN000023147).

### 2.1. HA-Coated Implant

**Figure 2:**
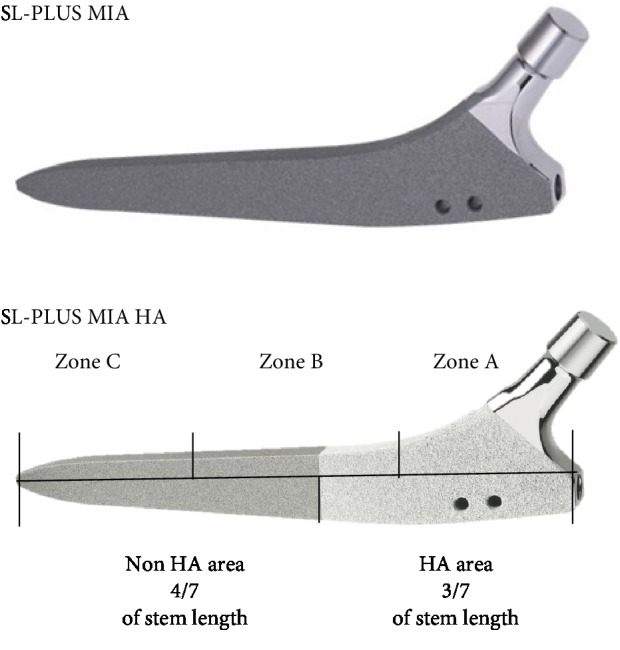
Profile of the hydroxyapatite coating on the SL-PLUS MIA HA stem. Zone A represents the HA-coated area, while zone C represents the non-HA-coated area. There are no areas with hydroxyapatite coating on the SL-PLUS MIA stem.

HA coating exists on three-sevenths of the stem length of the SL-PLUS MIA HA stem, covering its proximal region (Figure 2), while there is no area of HA coating on the SL-PLUS MIA stem.

### 2.2. Clinical Evaluation

The Harris hip score (HHS) was obtained before and 1 year after surgery.

### 2.3. BMD Measurement

**Figure 3:**
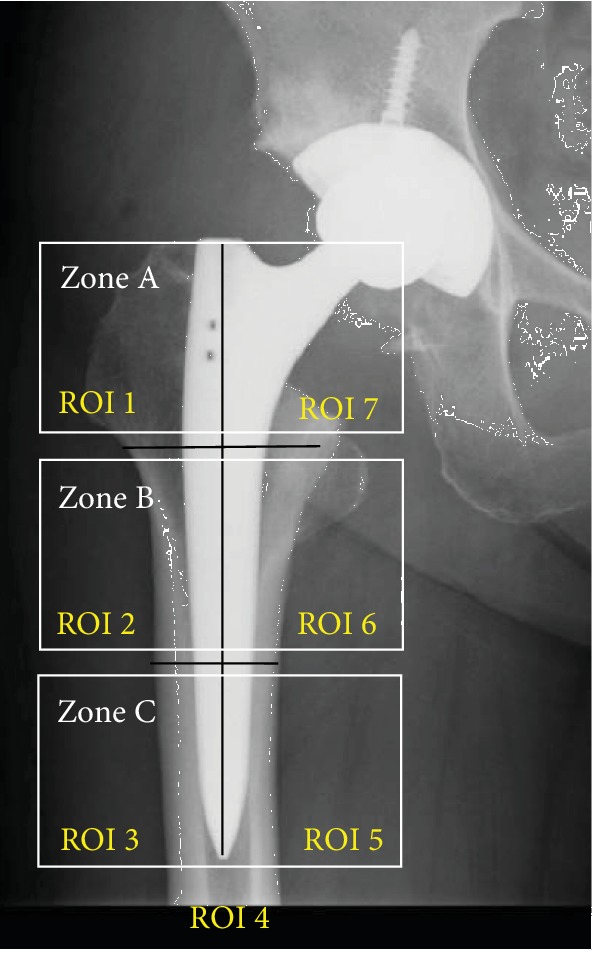
Definition of each region of interest (ROI) and zone. Zone A = ROI 1 + ROI 7, zone B = ROI 2 + ROI 6, and zone C = ROI 3 + ROI 5.

A Hologic Discovery system (Hologic, Inc., Waltham, MA) was used to perform the DEXA measurements to evaluate BMD. The DEXA measurements were performed at the end of the first postoperative week as a baseline reference, and then at 24 and 48 weeks after THA. Regions of interest (ROIs) were defined in accordance with the Gruen zones [19] (Figure 3). BMD change was calculated as the percentage change relative to the 1 week baseline and the evaluation of the BMD change at 48 weeks was the primary outcome of the current study.

### 2.4. PET Analysis

**Figure 4:**
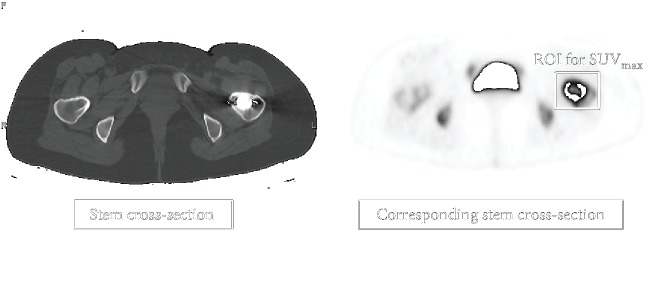
Measurement of the maximum standardized uptake value (SUV_max_) on PET/CT. The SUV_max_ was measured by setting a region of interest (ROI) around the prosthetic femoral stem in the axial image. The fused CT image was used to determine the level of the cross-section of the stem.

^18^F-fluoride PET imaging was performed 24 weeks after surgery using a Celesteion™ scanner (Toshiba Medical Systems Corporation, Tochigi, Japan), which yielded 32 horizontal cross-sections at 0.5 mm intervals. Patients were intravenously infused with ^18^F-fluoride (185 MBq/ml) dissolved in 10 ml of 0.9% saline and were then scanned 40 min later. The maximum standardized uptake values (SUV_max_) around the stems were calculated using the following formula: SUV_max_ = maximum radioactivity concentration in the tissue around the implant stem × attenuation correction ÷ (injected radioactivity dose ÷ body weight). To determine the exact anatomical location of the stem, the horizontal PET images were coregistered and fused with corresponding CT images. For each subject, the SUV_max_ was measured for every horizontal PET image that included a cross-section of the stem (Figure 4). SUV_max_ values for zones A and C (Figures 2 and 3) were determined by calculating the average of the SUV_max_ values from all the ROIs in each zone. The ROIs of each PET image were determined using the following algorithm. The PET image containing the cross-section of the tip of the proximal end of the stem is numbered “1,” and the following images are incrementally numbered until the PET image with the cross-section of the distal end of the stem appears; this number is referred to as *n*, because it is determined according to the length of each stem. Then, PET images from number 1 to *n*/3 are defined as zone A, images from number *n*/3 + 1 to 2*n*/3 belong to zone B, and images from number 2*n*/3 + 1 to *n* belong to zone C. Data from zone B were not used in the analysis, because this zone covers the transitional region between the HA-coated and non-HA regions (Figure 1).

### 2.5. Statistical Analysis

Unpaired Student's t *t*est was performed to evaluate the difference in HHS between the HA group and non-HA group. Unpaired Student's *t* test was also used to examine the significance of the differences in the BMD after 24 and 48 weeks after surgery between the HA group and non-HA group at all ROIs around the femoral prosthesis. The BMD values of zones A and C were calculated using the values of each of the ROIs: BMD for zone A = average BMD of ROIs 1 and 7 and BMD for zone C = average BMD of ROIs 3 and 5 (Figures 2 and 3) and between-group comparisons were performed using unpaired Student's *t* tests. The alpha level was set at 5%. To evaluate correlations between BMD and SUV_max_, Pearson's correlation coefficient was calculated between the BMD and SUV_max_ at 24 weeks after surgery. All statistical analyses were performed using SPSS, version 21.0 (IBM Corp., Armonk, NY, USA), and *the normality of data distribution for BMD and SUV_max_ was determined with Shapiro Wilk*.

## 3. Results

The average HHS improved from 58 ± 12 points before surgery to 93 ± 6.7 points postoperatively (*p* < 0.01). The average preoperative HHS for pain, function, deformity, and motion significantly improved. In the non-HA group, the mean postoperative HHS was 92 points, and in the HA group, the mean postoperative HHS was 93 points. There was no significant difference between the non-HA and HA groups.

**Figure 5:**
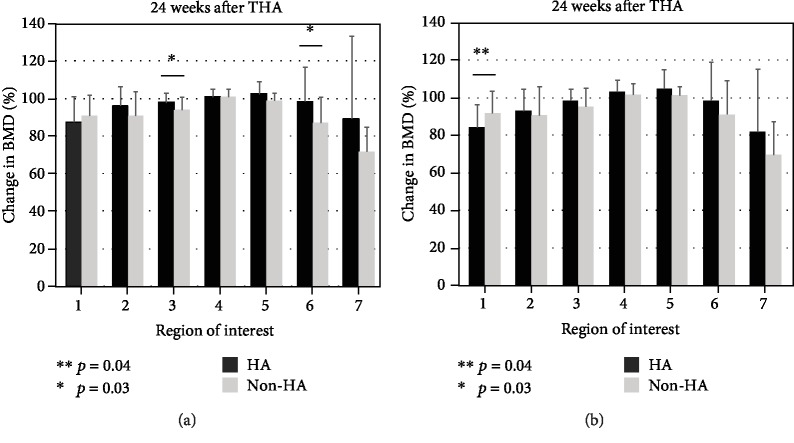
Changes in BMD in each region of interest at (a) 24 weeks and (b) 48 weeks, after surgery. BMD changes are calculated as percentages relative to the first week values. In chart (a), the BMD decreases in ROIs 3 and 6 in the HA group are significantly smaller than that in the non-HA group (*p* = 0.03). In chart (b), the BMD decrease in ROI 1 of the non-HA group was significantly smaller than that in the HA group (*p* = 0.04).

Figure 5 and Table 2 shows the periprosthetic relative BMD changes in all regions, for both the HA and non-HA groups. The BMD decreases in ROIs 3 and 6 in the HA group were significantly smaller than those in the non-HA group (*p* = 0.03) at 24 weeks after surgery, and the BMD decreases in ROIs 1 in the HA group were significantly smaller than those in the non-HA group (*p* = 0.04) at 48 weeks after surgery.

**Figure 6:**
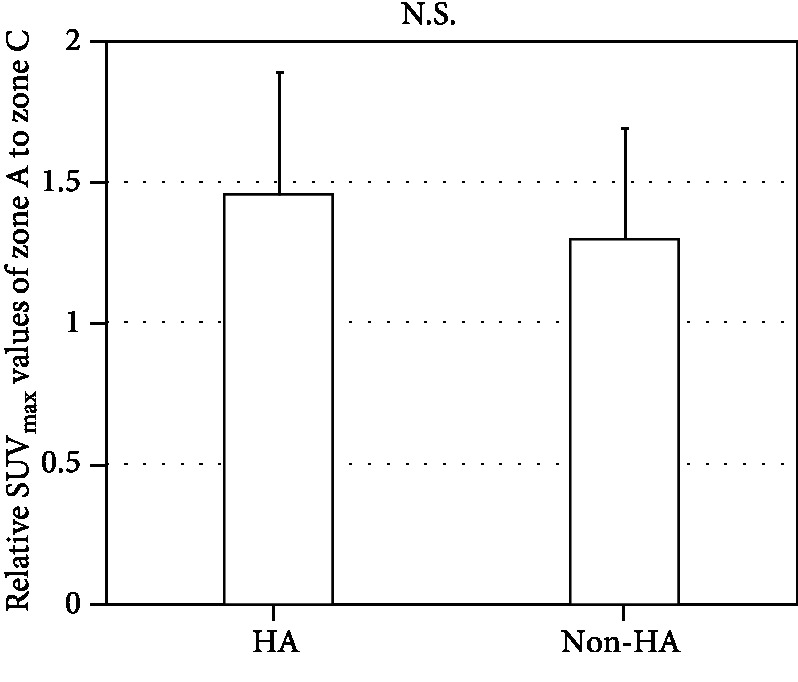
Difference in relative SUV_max_ between the HA and non-HA groups. SUV_max_ was described with mean and standard deviation. No significant difference was observed between the two groups (*p* = 0.35).

**Figure 7:**
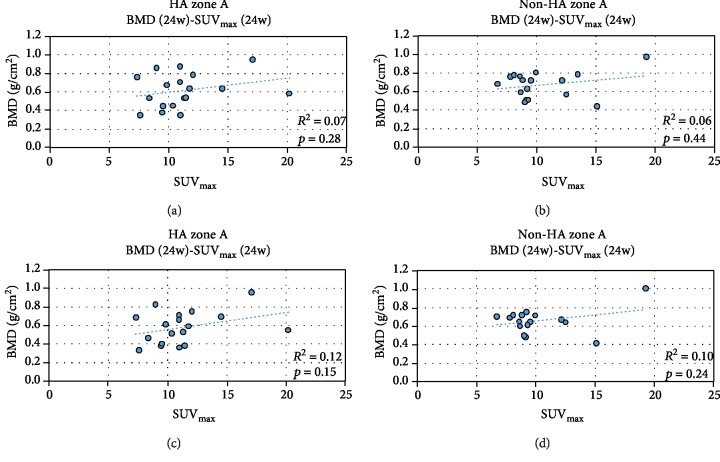
Relationships between BMD at 48 weeks and SUV_max_ at 24 weeks in zone A, with or without HA coating. No statistically significant correlations were observed between BMD and SUV_max_ values in zone A.

Figure 6 shows a comparison of the SUV_max_ values of zone A relative to zone C for the HA-coated stems and non-HA stems. The average relative SUV_max_ values were 1.45 for HA and 1.30 for non-HA. Although the average SUV_max_ value was higher in the HA group, the difference did not reach statistical significance (*p* = 0.35). Scatter plots of the relationships between SUV_max_ and BMD (24 or 48 weeks) in zone A are presented in Figure 7. No significant correlation was observed in either group.

## 4. Discussion

In this study, we investigated the effect of a HA coating on implants for THA using ^18^F-fluoride PET and DEXA. The HA coating had no significant effect on preventing a BMD decrease in ROI 7. However, the BMD decrease in ROI 6 in the HA group was significantly smaller than that in the non-HA group. The PET SUV_max_ values did not show any significant differences between the two groups.

Decreases in BMD around the femoral implant after THA have been observed in a number of previous studies [20, 21]. Particularly, in Zweymüller stems, bone absorption around the proximal area is due to stress shielding and cortical hypertrophy at the distal part [22, 23]. In the current study, similar to previous reports, the amount of decrease of the BMD was shown in ROI 1 or 7. These results were due to the achievement of metaphyseal-diaphyseal fixation of the Zweymüller stem. However, there was tendency of decrease of the BMD at ROIs 3 to 5. This might be due to the short follow-up period in the current study.

Although many factors are involved in BMD, one of the most important is the mechanical load around the implant [24–26] and its influence on bone metabolism. The application of coatings or surface modifications can also play a role [27, 28], as demonstrated by Geesink et al. when they developed the HA-coated implant to improve biological fixation [29] and demonstrated good long-term clinical results confirmed by radiographic evaluation [30]. Furthermore, mechanical and histological evaluations have revealed several positive effects of HA coating [10, 31]. Thus, the positive biological influence of a HA coating might be evident in terms of histology that showed osseointegration indicating bone remodeling in a relatively short period [32]; however, BMD loss around implants is still a problem, even with HA-coated implants [33].

**Figure 8:**
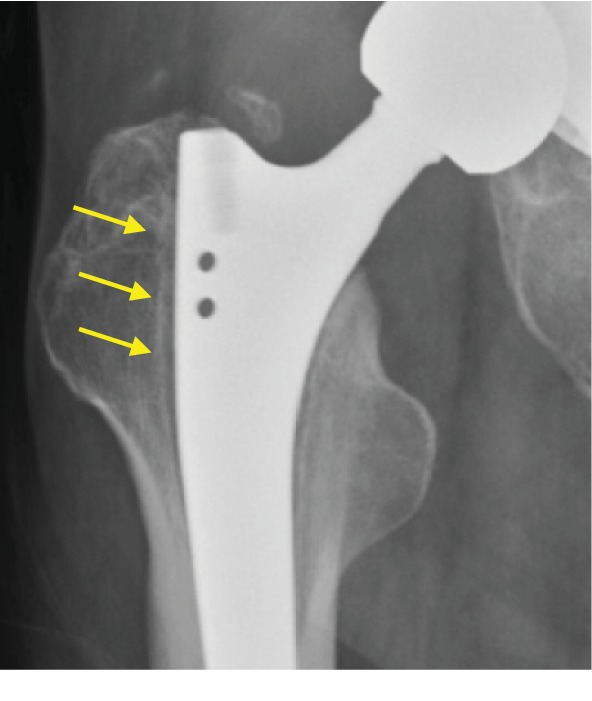
A 62-year-old female treated with non HA SL-PLUS MIA stem under the diagnosis of right hip osteoarthritis. On radiography, there was radiolucent line around proximal area accompanied by reactive line (yellow arrows).

In this study, we did show higher BMD in limited regions of ROIs 3 and 6 at 24 weeks in the HA group compared with that in the non-HA group; however, there was no significant difference at 48 weeks. *It is difficult to comprehend this observation*, *but one possible explanation is that the difference observed at 24 weeks is due to a transitional remodeling at the bone*-*implant interface.* We found that HA-coated stems showed no significant preventative effect on bone loss in the most severely affected region, i.e., ROI 7 in comparison with noncoated stems, although the average BMD loss in ROI 7 was 82% in the HA group and 69.7% in the non-HA group. Furthermore, the average BMD loss in ROI 1 in the non-HA group was significantly larger than that of the HA group. It seemed to be controversial; however, in our series, hips treated with non-HA stem showed radiolucent line with frequency in ROI 1 or 7. Since those with RLLs were accompanied by a reactive line around RLL (Figure 8), the development of a bone reactive line in cancellous bone in ROI 1 might induce higher BMD in the non-HA group compared to that in the HA group.

The investigation by Shu et al. showed that HA-coated surfaces increase osteoblast activity, differentiation, and mineralization, but that the coating decreases proliferation of osteoblasts [34]. If BMD is determined by both osteoblast mineralization and the numbers of osteoblast cells, a HA coating is likely to influence only the osteoblast mineralization factor. However, the PET uptake results in this study did not reveal any influence of the HA coating. Indeed, the effects of HA coatings on BMD outcomes are inconclusive between studies [35–39]; however, their clinical outcomes and thigh pain incidence are comparable to non-HA-coated stems [1].

Little is known about the quantitative changes in bone metabolism during osseointegration of uncemented femoral stems. It is not possible to determine these changes using conventional imaging and the metabolic state of the bone around the implant is still unknown. Although it is difficult to quantify osteoblastic activity around the implant on conventional imaging modalities, continuous advances in PET technology have enabled us to quantify periprosthetic osteoblastic activity [13], and the close relationship between HA and osteoblast dynamics has been clarified [34]. The mechanism for deposition of 18F-ions into the bone involves 18F ions passing from the plasma, through the extracellular fluid space, into the shell of bound water surrounding each crystal, and then onto the crystal surface and the interior of the crystal, as described by Blau et al. [40]. The SUV_max_ represents the quantity of HA crystal deposition due to osteoblasts, which is in turn determined by osteoblastic activity or the number of cells. Therefore, we speculated that a HA-coated stem would substantially influence the degree of PET uptake. In the contrary, we could not identify any influence of the HA coating in terms of the SUV_max_, at least within the first postoperative year. Moreover, we identified a significant decrease in BMD in ROI 1 in the HA group compared with that in the non-HA group. This fact needs to be addressed in further investigations that include radiographic analysis.

No significant correlation was observed between BMD and SUV_max_, as shown in Figure 7. We had to categorize femoral zone to A, B, and C rather than ROI 1~7 for measuring SUV_max_, because we need to measure SUV_max_ in each axial slice. Therefore, the actual correlation between BMD and SUV_max_ in terms of each ROI was not evaluated. The BMD change around the stem is affected by the mechanical stress properties of the stem [24]. Although we presumed that bone metabolic activity (i.e., osteoblast activity) has a great influence on BMD change, changes in bone metabolic activity were not detected by the uptake of ^18^F-fluoride on PET. In zone A, where mechanical stress is relatively weak [24], distal mechanical interlocking causes the larger stress in the metaphyseal-diaphyseal area. Consequently, consistent with Wolf's law, the bone within the proximal femur will be resorbed regardless of stem surface finish.

There are several limitations in this study. The major limitation of this study is the relatively short observation period. Although BMD change might be observed after longer follow-up, Nishi et al. reported a large part of the bone remodeling after cementless hip arthroplasty ceases, occuring within 1 year postoperatively; thereafter, the BMD change appeared to be stabilized. ^18^F-fluoride PET imaging was performed only once within the time period of 48 weeks. Additional time points for ^18^F-fluoride PET imaging may reveal different results. Ullmark et al. reported that ^18^F-fluoride PET uptake around femoral prosthesis continued to increase after about 4 weeks after THA then decreased 1 year after surgery [16]. Another major limitation is that whether ^18^F-fluoride PET truly monitored bone turnover around the femoral stem. In this regard, Ullmark used ^18^F-fluoride PET not only for detecting aseptic loosening [15] but also for evaluating mineralization of bone in the femur adjacent to uncemented stems following THA [16, 17]. Although a number of subjects dropped out, it was not possible to enroll more subjects during the study period because there was only limited ^18^F-fluoride PET availability. In the manufactural design, the HA coating is limited to the proximal part of the stem, which is affected by mechanical stress distribution. HA coatings on different parts of the stem, or different stem designs, may lead to different results.

## 5. Conclusion

In this study, the Zweymüller stem with HA coating on the proximal area showed only a limited influence on the prevention of BMD loss around implants, at least during the first year postsurgery. HA coating at the proximal area of the Zweymüller stem did not increase the SUV_max_ of ^18^F-fluoride PET compared with non-HA implants. There was no significant correlation between the SUV_max_ of ^18^F-fluoride PET and BMD, with or without a HA coating.

## Figures and Tables

**Table 1:** Demographic data.

	HA	Non-HA	*p* value
Number of hips	32	20	
Gender (female/male)	27/5	19/1	0.24^∗^
Osteoarthritis/osteonecrosis	29/3	17/3	0.54^†^
Average age	65 ± 12	65 ± 10	0.66^∗^
Average BMI	23.7 ± 3.8	24.7 ± 5.5	0.44^∗^
Harris hip score	54 ± 15	64 ± 9	0.09^∗^

^∗^Unpaired Student's *t* test. ^†^Chi-square test.

**Table 2:** The results of the BMD at 24 and 48 weeks after surgery.

		ROI 1	ROI 2	ROI 3	ROI 4	ROI 5	ROI 6	ROI 7
24 weeks	HA	87.4	96.3	97.9	101	102.4	98.4	89.4
	Non-HA	91.4	90.8	93.8	100.5	99.1	87.5	72.2

	*p* value	N.S.	N.S.	0.03	N.S.	N.S.	0.03	N.S.

48 weeks	HA	84.3	93	98.3	103	104.6	98.3	82
	Non-HA	91.9	90.6	95.2	101.8	101.1	91.1	69.7

	*p* value	0.04	N.S.	N.S.	N.S.	N.S.	N.S.	N.S.

N.S.: not significant.

## Data Availability

The data used to support the findings of this study are included within the article.

## Abbreviations

HA: Hydroxyapatite
BMD: Bone mineral density
THA: Total hip arthroplasty
DEXA: Dual-energy X-ray absorptiometry
ROI: Region of interest
PET: Positron emission tomography
SUV_max_: The maximum standardized uptake values.
